# Proteomics reveals drastic increase of extracellular matrix proteins collagen and dermatopontin in the aged mdx diaphragm model of Duchenne muscular dystrophy

**DOI:** 10.3892/ijmm.2012.1006

**Published:** 2012-05-18

**Authors:** STEVEN CARBERRY, MARGIT ZWEYER, DIETER SWANDULLA, KAY OHLENDIECK

**Affiliations:** 1 Department of Biology, National University of Ireland, Maynooth, Kildare, Republic of Ireland;; 2 Department of Physiology II, University of Bonn, D-53115 Bonn, Germany

**Keywords:** collagen, dermatopontin, diaphragm, muscular dystrophy, proteomics

## Abstract

Duchenne muscular dystrophy is a lethal genetic disease of childhood caused by primary abnormalities in the gene coding for the membrane cytoskeletal protein dystrophin. The mdx mouse is an established animal model of various aspects of X-linked muscular dystrophy and is widely used for studying fundamental mechanisms of dystrophinopathy and testing novel therapeutic approaches to treat one of the most frequent gender-specific diseases in humans. In order to determine global changes in the muscle proteome with the progressive deterioration of mdx tissue with age, we have characterized diaphragm muscle from mdx mice at three ages (8-weeks, 12-months and 22-months) using mass spectrometry-based proteomics. Altered expression levels in diaphragm of 8-week vs. 22-month mice were shown to occur in 11 muscle-associated proteins. Aging in the mdx diaphragm seems to be associated with a drastic increase in the extracellular matrix proteins, collagen and dermatopontin, the molecular chaperone αB-crystallin, and the intermediate filament protein vimentin, suggesting increased accumulation of connective tissue, an enhanced cellular stress response and compensatory stabilization of the weakened membrane cytoskeleton. These proteomic findings establish the aged mdx diaphragm as an excellent model system for studying secondary effects of dystrophin deficiency in skeletal muscle tissue.

## Introduction

The largest human gene spans 2.5 Mb of the X-chromosome and encodes the membrane cytoskeletal protein dystrophin of 427 kDa (1). Primary abnormalities in the dystrophin gene lead to a functional absence of the full-length Dp427 isoform and trigger Duchenne muscular dystrophy, a progressive neuromuscular disease of childhood (2). The muscular dystrophy X-linked (mdx) mouse is an established animal model of various aspects of X-linked muscular dystrophy and is widely used for studying fundamental mechanisms of dystrophinopathy and testing novel therapeutic approaches to treat one of the most frequent gender-specific diseases in humans (3). A single base substitution within exon 23 of the dystrophin gene causes premature termination of the dystrophin polypeptide chain in mdx mice (4). Although most individual muscles in the mdx mouse do not represent a perfect replica of the fiber wasting pathology observed in the highly progressive etiology of Duchenne muscular dystrophy, certain muscle types show many molecular and cellular alterations that are characteristic of dystrophinopathy.

The mdx mouse shows i) a loss of the sarcolemmal dystrophin isoform Dp427 and a drastic reduction in dystrophin-associated glycoproteins in contractile cells (5); ii) elevated levels of serum creatine kinase indicative of reduced muscle fiber integrity (6); iii) a varying degree of muscle degeneration ranging from minimal effects in extraocular and laryngeal muscle (7) to segmental necrosis in limb muscle (8) to severe fiber wasting in diaphragm muscle (9); iv) a high susceptibility to osmotic shock (10) or stretch-induced injury (11); and v) abnormal calcium-handling (12) including elevated cytosolic Ca^2+^-levels (13). These genetic, biochemical, physiological and cell biological abnormalities have established the mdx mouse as a suitable, albeit not precise, genocopy and phenocopy of X-linked muscular dystrophy (3). The mdx model system has been widely used for testing new therapeutic approaches, including myoblast transfer therapy (14), gene therapy (15), exon skipping therapy (16) and pharmacological intervention (17) and is thus a crucial tool for the future establishment of new treatment options (18). In contrast to considerable phenotypic differences between young mdx muscle and human dystrophic specimens, a large number of studies have demonstrated that mdx muscle tissue progressively deteriorates with age and more closely resembles the human pathology (19).

The age-related mdx pathology includes progressive motor weakness (20), loss of myofibers and replacement by extensive connective tissue (21–23), the presence of branched fibers that exhibit mechanical weakening of the sarcolemma (24), a reduced life span and increased susceptibility to spontaneous rhabdomyosarcoma (25), impaired functional and structural recovery after injury (26), and a decline in regenerative potential and alterations in the crucial mTOR signaling pathway, which is of central importance for muscle development, muscle regeneration, and muscle growth in response to nutrients, growth factors and exercise (27). Thus, since senescent mdx muscle tissues appear to represent a more suitable dystrophic phenotype, it was of interest to determine global changes in the protein complement during the natural aging process of mdx muscle tissue. This report shows the findings of a comparative proteomic analysis of severely affected diaphragm muscle from 8-week, 12-month and 22-month dystrophic specimens.

## Materials and methods

### Chemicals and materials

Materials and electrophoresis-grade chemicals for the proteomic analysis of muscle proteins were purchased from Amersham Biosciences/GE Healthcare (Little Chalfont, UK). For protein digestion, sequencing grade-modified trypsin was obtained from Promega (Madison, WI). Chemiluminescence substrate and protease inhibitors were from Roche Diagnostics (Mannheim, Germany). Primary antibody to collagen VI and secondary peroxidase-conjugated antibodies were from Abcam (Cambridge, UK) and Chemicon International (Temecula, CA), respectively. All other chemicals used were of analytical grade and were purchased from Sigma Chemical Co. (Dorset, UK).

### Preparation of crude muscle extracts from aged mdx mice

The mdx mouse is missing the membrane cytoskeletal protein Dp427 due to a point mutation in the dystrophin gene (4) and the severely affected mdx diaphragm muscle represents an established animal model of Duchenne muscular dystrophy (9). Dystrophic diaphragm muscle from 8-week, 12-month and 22-month mdx mice and normal tissues from age-matched C57 mice were obtained from the bioresource unit of the University of Bonn (26). Mice were kept under standard conditions and all procedures were performed in accordance with German guidelines on the use of animals for scientific experiments. Animals were sacrificed by cervical dislocation and muscle tissues were quickly removed and quick-frozen in liquid nitrogen. For the proteomic analysis of mdx tissue, specimens were shipped to Ireland on dry ice and stored at −80°C prior to usage. In order to obtain diaphragm protein extracts, 4 dystrophic muscle specimens from each age group were pulverized by grinding tissue pieces in liquid nitrogen using a mortar and pestle. Ground muscle powder was solubilized in lysis buffer with the ratio of 100 mg wet weight to 1 ml lysis buffer [7 M urea, 2 M thiourea, 4% CHAPS, 2% IPG buffer pH 3–10, 2% (w/v) DTT]. To prevent excess protein degradation, the lysis buffer was supplemented with a freshly prepared protease inhibitor cocktail (28). Following gentle rocking for 60 min, suspensions were centrifuged at 4°C for 20 min at 20,000 x g and the protein concentration determined (29).

### Fluorescence gel electrophoretic analysis

For the separation of individual muscle protein species, two-dimensional gel electrophoresis was carried out by previously optimized methodology using first dimension isoelectric focusing with pH 3–10 strips and second dimension slab gel electrophoresis with 500 *μ*g protein/ gel (28–30). Twelve slab gels were run in parallel at 0.5 W/gel for 60 min and then 15 W/gel until the blue dye front had disappeared from the bottom of the gel. Post-electrophoretic staining for the total protein profile was performed with the fluorescent dye ruthenium II tris bathophenanthroline disulfonate (RuBPs). A stock solution of RuBPs dye was prepared as described previously by Rabilloud *et al* (31). Following fixation for 30 min in 30% ethanol and 10% acetic acid, gels were washed 3 times for 30 min in 20% ethanol and then stained for 6 h in 20% (v/v) ethanol containing 2 *μ*M of ruthenium chelate. Gels were re-equilibrated twice for 10 min in distilled water and destained overnight in 40% ethanol and 10% acetic acid prior to imaging (32). Fluorescently labelled proteins were visualized using a Typhoon Trio variable mode imager (Amersham Biosciences/ GE Healthcare). Gel analysis was performed with Progenesis 2D analysis software (Nonlinear Dynamics, Newcastle upon Tyne, UK) and protein spots with a significant change in abundance were identified by mass spectrometry.

### Mass spectrometric identification of muscle-associated proteins

Protein identification was performed with 2D protein spots from Coomassie-stained pick gels, following counter staining of RuBPs-labelled analytical gels. Electrospray ionization LC-MS/MS analysis was carried out as previously described in detail (29). Previously standardized in-gel tryptic digestion protocols were employed for the reproducible generation of peptides for mass spectrometric analysis on a Model 6340 Ion Trap LC/MS apparatus from Agilent Technologies (Santa Clara, CA). Database searches were carried out using Mascot MS/ MS Ion search. Criterion for each search was set at i) species Mus musculus, ii) two missed cleavages by trypsin, iii) variable modification: oxidation of methioine, iv) fixed modification: carboxymethylation of cysteines and v) mass tolerance of precursor ions ±2 Da and product ions ±1 Da. Verification of key proteomic findings was carried out by comparative immunoblot analysis (28).

## Results

### Gel electrophoretic analysis of aged mdx diaphragm muscle

**Figure 1:**
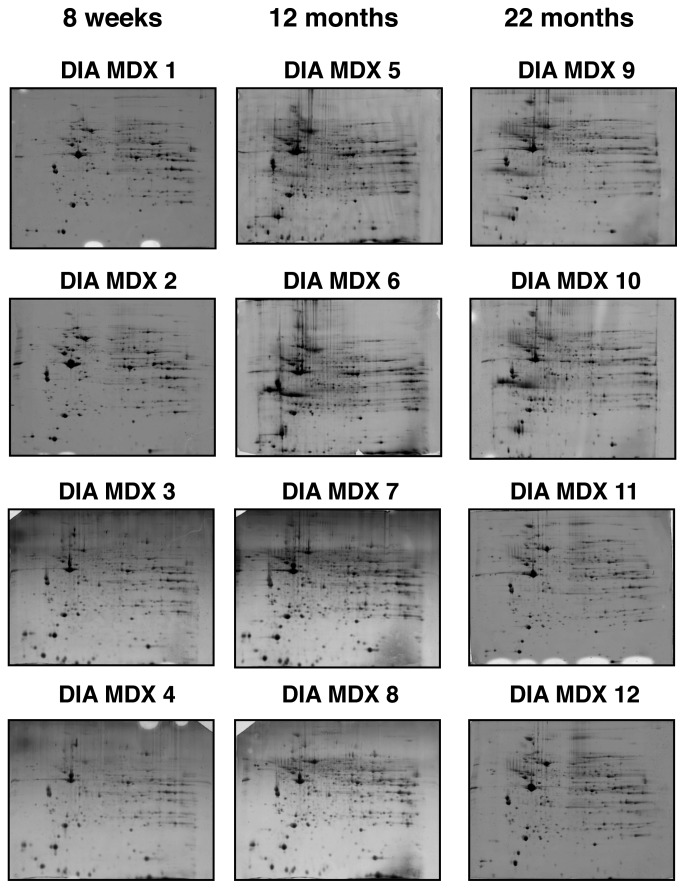
Two-dimensional gel electrophoretic analysis of aged mdx diaphragm muscle. Shown are RuBPs-stained gels of total extracts from 8-week (DIA MDX 1–4), 12-month (DIA MDX 5-8) and 22-month (DIA MDX 9–12) muscle. Fluorescent images are shown for the pH 3–10 range.

Fluorescence high-resolution 2D gel electrophoresis in combination with MS analysis was used to detect potential differences in aging-related protein expression patterns in severely dystrophic diaphragm muscle from mdx mice. As summarized in Fig. 1, gels representing 4 biological repeats of 8-week, 12-month and 22-month mdx diaphragm muscle were analyzed. The overall 2D spot patterns of differently aged dystrophic preparations were relatively comparable; requiring therefore detailed denitometric analyses for the determination of significant differences in individual muscle proteins. With the help of a Typhoon Trio variable imager and Progenesis 2-D analysis software, individual muscle proteomes separated on 12 different gels were compared. Panels DIA MDX 1–4, DIA MDX 5–8 and DIA MDX 9–12 represent 8-week, 12-month and 22-month diaphragm muscle preparations, respectively. The detailed proteomic survey of dystrophic diaphragm muscle tissue identified distinct changes in a variety of protein species.

### Proteomic analysis of protein alterations in aged mdx diaphragm muscle

**Figure 2:**
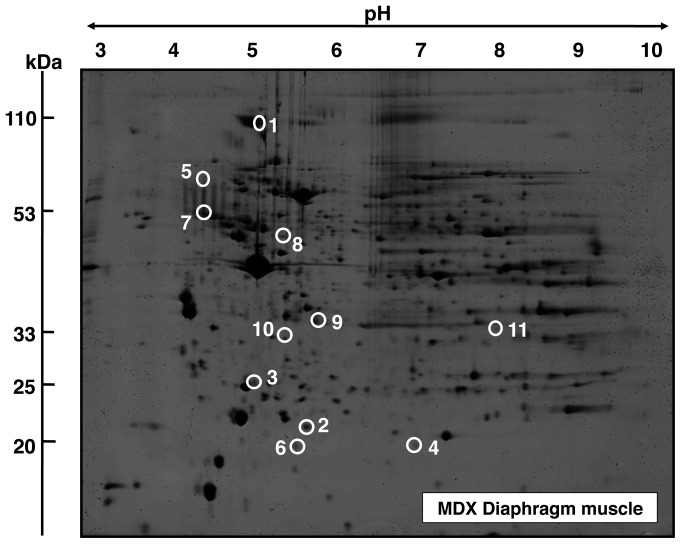
Fluorescence gel electrophoretic analysis of mdx diaphragm. Shown is a representative RuBPs-labeled master gel of crude tissue extracts from mdx diaphragm. Protein spots with an age-related change in expression levels are marked by circles and are numbered 1 to 11. See Table I for the mass spectrometric identification of individual muscle-associated proteins. The pH-values of the first dimension gel system and molecular mass standards of the second dimension are indicated on the top and on the left of the panels, respectively.

A representative fluorescent 2D master gel of mdx diaphragm muscle is shown in Fig. 2. The overall number and degree of age-related changes was striking in diaphragm muscle. Skeletal muscle proteins that exhibited significant alterations in expression levels are marked by circles and are numbered 1 to 11 in 2D gels of diaphragm muscle. The mass spectrometric identification of these altered protein species is catalogued in Table I. Listed are the names of the identified muscle-associated proteins, their international accession number, p*I*-values, their relative molecular masses, the number of matched peptide sequences, percentage sequence coverage, Mascot scores, and fold-change of individual proteins affected in dystrophin-deficient tissue during aging.

### Mass spectrometrically identified proteins with an altered abundance in mdx diaphragm muscle

Protein species with a changed concentration in mdx diaphragm muscle ranged in molecular mass from 20 kDa (αB-crystallin) to 110 kDa (collagen) and covered a p*I*-range from 4.7 (dermatopontin) to 8.6 (myozenin). As presented in Fig. 2 and Table I, an increased concentration was established for the α-1(VI) chain of collagen (spot 1), the extracellular matrix protein dermatopontin (spot 2), the enzyme ubiquitin carboxyl-terminal hydrolase (spot 3), the small heat shock protein αB-crystallin (spot 4), α-2 actinin (spot 5), ferritin heavy chain (spot 6), vimentin (spot 7), the γ chain of fibrinogen (spot 8) mimecan (spot 9) and apolipoprotein E (spot 10). Spot 11 representing myozenin was shown to be decreased in aged mdx diaphragm muscle.

### Immunoblot analysis of collagen in aged mdx diaphragm muscle

**Figure 3:**
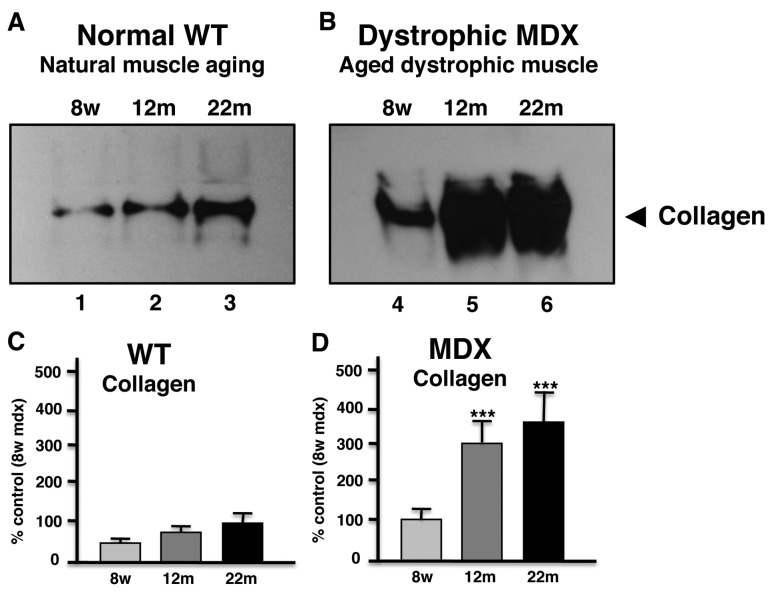
Immunoblot analysis of collagen in normal vs. mdx diaphragm muscle during aging. Shown are representative immunoblots with expanded views of antibody-decorated collagen bands. Lanes 1–3 and lanes 4–6 represent 8-week, 12-month and 22-month normal wild-type (A) vs. dystrophic mdx (B) diaphragm muscle, respectively. (C and D) The graphical representation of the immunoblotting of collagen in normal vs. mdx preparations (n=5; ^***^P<0.001; unpaired t-test), respectively.

In order to independently verify the most drastic alteration in aged mdx diaphragm as revealed by proteomics, comparative immunoblotting was used. As shown in Fig. 3, immunodecoration of gel electrophoretically separated normal and mdx diaphragm of varying age showed a general increase of collagen in 12-month and 22-month muscle preparations. However, aged dystrophic mdx diaphragm muscle exhibited a significantly higher increase in collagen as compared to aged normal muscle.

## Discussion

Animal models that mimic neuromuscular disorders play a crucial role in basic and applied myology. Naturally occurring or genetically engineered model systems are widely used for studying fundamental aspects of molecular and cellular pathogenesis, as well as the evaluation of novel therapeutic approaches (33). Ideally, an animal model of a genetic disorder should: i) exhibit similar primary abnormalities and secondary downstream alterations as seen in the corresponding human disease; ii) closely develop most of the multifactorial features observed in complex human pathologies; iii) resemble the pathogenesis of the human disease in onset, progression and severity; iv) show sufficient similarities to human metabolism, physiology and immune responses so that these biological factors do not have a major differentiating influence on disease progression in animal models vs. patients; v) is easy to breed and house at a reasonable cost; vi) be suitable for genetic manipulations and the facilitation of physiological and surgical procedures; and vii) be large enough to yield sufficient amounts of tissue specimens for extended biological analyses (34). Since an important bioethical objection with respect to the humane and responsible use of animal models in biomedical research is often associated with the usage of larger animals, small rodents are the most frequently used alternatives as genetic model systems. In the case of one the most progressive genetic disease of the neuromuscular system, Duchenne muscular dystrophy, the naturally occurring dystrophic mdx mouse has been employed in a large number of studies (3,35).

In analogy to findings of previous mass spectrometry-based proteomic studies of contractile tissues from young or mature mdx mice (36) which included hindlimb muscle (37–39), extraocular muscle (30), diaphragm (16,28,40) and heart (29,41), we have here carried out a comparative proteomic survey of diaphragm muscle from 8-week, 12-month and 22-month dystrophic specimens. Dystrophic diaphragm muscle showed altered expression levels in 11 proteins during skeletal muscle aging. Changes in the diaphragm proteome from aged mdx mice suggested elevated levels of fibrosis, an intensified stress response and an increase in cytoskeletal elements possibly compensating the lack of dystrophin. These proteomic findings agree with the idea of more extensively perturbed protein expression levels in dystrophin-deficient diaphragm fibers as compared to other mdx muscle systems (36).

The approximately 6-fold increase of collagen and dermatopontin in senescent mdx diaphragm muscle are important proteomic findings and reflect the dystrophic status of this muscle type. The dramatic increase of collagen in mdx diaphragm was clearly confirmed by immunoblot analysis, verifying the findings from the mass spectrometric investigation presented here. Although it is clearly established that collagen levels increase in the skeletal muscle extracellular matrix during the natural aging process (42), the dystrophic phenotype shows an exacerbated age-related accumulation of collagen α-1(VI) chain. Collagen is the main protein component of connective tissue and is especially enriched in the endomysium of skeletal muscles. Increased collagen protein levels agree with previously reported greater amounts of collagen mRNA in the mdx diaphragm (43) and support the idea of severe fibrosis in the mdx diaphragm (44,45). The non-collagenous extracellular matrix protein dermatopontin is involved in cell-matrix interactions and matrix assembly (46) and also named tyrosine-rich acidic matrix protein (TRAMP) (47). TRAMP appears to regulate interactions of TGF-β, decorin and fibronectin (48). Its greater abundance in mdx diaphragm is probably due to increased demands for collagen matrix organization within dystrophic muscle tissues. Increased levels of fibrinogen in aged mdx diaphragm, as shown here by proteomics, agree with a previous study by Vidal *et al* (49). Fibrinogen seems to play a key role in fibrosis via a TGF-β/alternative macrophage activation pathway in dystrophinopathy (49).

The increase of αB-crystallin and vimentin suggests an increased cellular stress response and an upregulation of cytoskeletal elements in dystrophic muscle, respectively, which agrees with previous proteomic studies (28). In the future, it will be interesting to define the potential pathobiochemical role of newly identified biomarkers of muscular dystrophy in aged mdx diaphragm muscle, including the deubiqutinating enzyme ubiquitin carboxyl-terminal hydrolase, the microfilament protein actinin and its binding protein myozenin, the intracellular iron storage component ferritin, the connective tissue protein mimecan, and the triglyceride transporter apolipoprotein E. Overall, the proteomic results presented here suggest that the aged mdx diaphragm, which exhibits severe respiratory impairment following fibrosis (50), is a highly suitable model system for studying the molecular pathogenesis of Duchenne muscular dystrophy. Collagen and dermatopontin should be considered as suitable biomarker candidates for evaluating the degree of fibrosis and tissue scaring in dystrophinopathy.

## Figures and Tables

**Table I:** The identified proteins that exhibit a drastic change in abundance during aging of the severely dystrophic mdx diaphragm muscle.

Spot no.	Protein name	Protein accession no.	Isoelectric point, p*I*	Molecular mass (Da)	Peptides, n	Coverage (%)	Mascot score	Fold-change 8 w-12 m	Fold-change 8 w-22 m
1	Collagen α-1(VI) chain	NP034063	5.20	109,582	12	15	259	3.6	6.3
2	Dermatopontin	NP062733	4.70	24,559	4	21	122	5.4	6.1
3	Ubiquitin carboxyl-terminal hydrolase UCHL1	AAD51029	5.33	25,170	4	30	83	3.5	4.1
4	αB-crystallin	NP034094	6.76	20,056	7	38	113	3.7	4
5	Actinin, α2	AAK64510	5.34	104,447	4	5	196	4.3	3.6
6	Ferritin heavy chain	NP034369	5.53	21,227	3	18	50	2.6	2.6
7	Vimentin	CAA39807	5.06	53,747	14	37	140	2.5	2.5
8	Fibrinogen, γ chain	NP598623	5.54	50,056	4	12	65	1.9	2.5
9	Mimecan	NP032786	5.52	34,339	5	19	293	1.8	2.2
10	Apolipoprotein E	AAA37252	5.82	33,206	8	32	169	2	1.5
11	Myozenin-1	NP067483	8.57	31,438	9	54	174	0.3	0.3

## Abbreviations

MS: mass spectrometry;
mdx: muscular dystrophy X-linked;
2D: two-dimensional;
RuBPs: ruthenium tris bathophenanthroline disulfonate
